# Tris{4-[(2*H*-tetra­zol-5-yl)meth­yl]morpholinium} dodeca­tungstophosphate hexa­hydrate

**DOI:** 10.1107/S1600536811003734

**Published:** 2011-02-05

**Authors:** Babak Feizyzadeh, Masoud Mirzaei, Hossein Eshtiagh-Hosseini, Ahmad Gholizadeh

**Affiliations:** a Department of Chemistry, Islamic Azad University, Quchan Branch, Quchan, Iran; b Department of Chemistry, School of Sciences, Ferdowsi University of Mashhad, Mashhad 917791436, Iran; c Department of Chemistry, Islamic Azad University, North Tehran Branch, Tehran, Iran

## Abstract

The title heteropolyoxidotungstate-based inorganic–organic hybrid material, (C_6_H_12_N_5_O)_3_[W_12_(PO_4_)O_36_]·6H_2_O, consists of one α-Keggin-type [W_12_(PO_4_)O_36_]^3−^ polyoxidometalate anion (POM), three crystallographically independent 4-[(2*H*-tetra­zol-5-yl)meth­yl]morpholinium cations and six water mol­ecules of crystallization. The morpholine ring of the cation adopts a chair conformation. The anion shows characteristic features with respect to bond lengths and angles. An extensive network of N—H⋯O, N—H⋯N, O—H⋯O and O—H⋯N hydrogen-bonding inter­actions between the organic cations, inorganic anion and the crystal water mol­ecules lead to a three-dimensional structure. Moreover, six uncoordinated water mol­ecules increase the number of hydrogen bonds in the network and lead to the formation of (H_2_O)_∞_ clusters.

## Related literature

For other inorganic–organic hybrid materials based on polyoxidometalates with organic cations, see: Alizadeh *et al.* (2008*a* ▶,*b* ▶); Nikpour *et al.* (2009 ▶, 2010 ▶). For details of (H_2_O)_*n*_ cluster analysis, see: Aghabozorg *et al.* (2010 ▶). For background to pseudopolymorphism, see: Desiraju (2003 ▶).
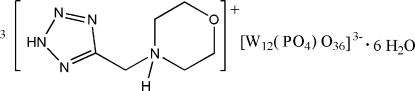


## Experimental

### 

#### Crystal data

(C_6_H_12_N_5_O)_3_[W_12_(PO_4_)O_36_]·6H_2_O
*M* *_r_* = 3495.88
Orthorhombic, 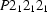

*a* = 14.616 (3) Å
*b* = 15.213 (3) Å
*c* = 26.735 (6) Å
*V* = 5944 (2) Å^3^
*Z* = 4
Mo *K*α radiation
μ = 23.27 mm^−1^
*T* = 100 K
0.12 × 0.11 × 0.06 mm

#### Data collection

Bruker APEXII CCD area-detector diffractometer
Absorption correction: multi-scan (*SADABS*; Bruker, 2005 ▶) *T*_min_ = 0.078, *T*_max_ = 0.246
59635 measured reflections
12321 independent reflections
9617 reflections with *I* > 2σ(*I*)
*R*_int_ = 0.159

#### Refinement

*R*[*F* ^2^ > 2σ(*F* ^2^)] = 0.052
*wR*(*F* ^2^) = 0.104
*S* = 1.00
12321 reflections
446 parameters
6 restraints
H-atom parameters constrained
Δρ_max_ = 2.80 e Å^−3^
Δρ_min_ = −2.72 e Å^−3^
Absolute structure: Flack (1983 ▶), 5544 Friedel pairs
Flack parameter: −0.041 (19)

### 

Data collection: *APEX2* (Bruker, 2005 ▶); cell refinement: *SAINT* (Bruker, 2005 ▶); data reduction: *SAINT*; program(s) used to solve structure: *SHELXTL* (Sheldrick, 2008 ▶); program(s) used to refine structure: *SHELXTL*; molecular graphics: *SHELXTL*; software used to prepare material for publication: *publCIF* (Westrip, 2010 ▶).

## Supplementary Material

Crystal structure: contains datablocks I, global. DOI: 10.1107/S1600536811003734/wm2435sup1.cif

Structure factors: contains datablocks I. DOI: 10.1107/S1600536811003734/wm2435Isup2.hkl

Additional supplementary materials:  crystallographic information; 3D view; checkCIF report

## Figures and Tables

**Table 1:** Hydrogen-bond geometry (Å, °)

*D*—H⋯*A*	*D*—H	H⋯*A*	*D*⋯*A*	*D*—H⋯*A*
N4*A*—H4*A*⋯O2*W*	0.88	1.90	2.77 (2)	173
N5*A*—H5*A*⋯O5*W*	0.90	1.88	2.76 (3)	166
N4*B*—H4*B*⋯O1*W*^i^	0.88	1.84	2.71 (2)	168
N5*B*—H5*B*⋯N2*A*^ii^	0.90	2.31	2.97 (2)	130
N4*C*—H4*C*⋯O2*W*	0.88	2.09	2.87 (3)	149
N5*C*—H5*C*⋯O4*W*	0.87	2.01	2.71 (2)	137
O1*W*—H1*W*⋯O3*C*^iii^	0.85	2.01	2.84 (2)	164
O1*W*—H2*W*⋯O3	0.85	1.92	2.77 (2)	180
O2*W*—H3W⋯O11*T*^iv^	0.85	2.43	2.87 (2)	113
O2*W*—H4W⋯O9*T*^v^	0.85	2.18	2.96 (2)	153
O2*W*—H4W⋯O2^vi^	0.85	2.54	3.06 (2)	121
O3*W*—H5*W*⋯N1*A*^ii^	0.85	2.41	3.03 (3)	130
O3*W*—H6*W*⋯N2*C*	0.85	2.14	2.79 (3)	134
O4*W*—H7*W*⋯O7*T*	0.85	2.11	2.96 (2)	180
O4*W*—H8*W*⋯O6*W*^vii^	0.85	2.00	2.82 (2)	161
O5*W*—H9*W*⋯O2*W*	0.85	1.99	2.82 (2)	164
O5*W*—H10*W*⋯O6*W*	0.85	1.93	2.78 (2)	178
O6*W*—H11W⋯N2*B*	0.85	2.11	2.85 (2)	146
O6*W*—H12W⋯O2*T*^v^	0.85	2.25	2.91 (2)	134
O6*W*—H12W⋯O1*W*^vi^	0.85	2.44	3.11 (2)	137

Symmetry codes: (i) 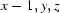; (ii) 
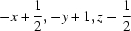
; (iii) 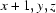; (iv) 
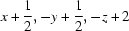
; (v) 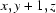; (vi) 
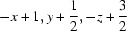
; (vii) 
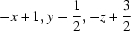
.

**Fig. 1.**
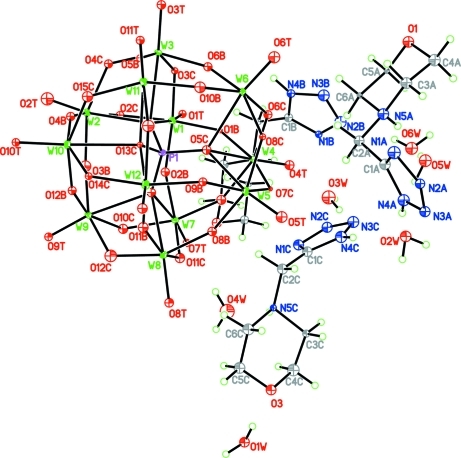
The asymmetric unit of [C6H12N5O]3[W12(PO4)O36].6H2O, with displacement ellipsoids drawn at the 50% probability level.

**Fig. 2.**
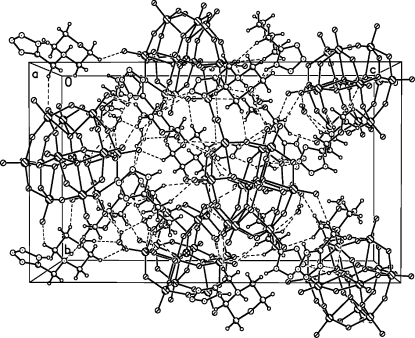
The crystal packing of (I) in a projection along a, emphasizing the three-dimensional H-bonded network (dashed lines).

## supplementary crystallographic information

### Comment

In continuation of our previous studies of inorganic-organic hybrid materials based on polyoxidometalates (Alizadeh *et al.*, 2008*a*,*b*; Nikpour *et al.*, 2009), we report here on the structure of the title compound, [C_6_H_12_N_5_O]_3_[W_12_(PO_4_)O_36_]^.^6H_2_O, (I), as a pseudopolymorph (different number of crystal water molecules; Desiraju, 2003) of [C_6_H_12_N_5_O]_3_[W_12_(PO_4_)O_36_]^.^5H_2_O  as reported by us previously (Nikpour *et al.*, 2010).

The structure of (I) consists of one discrete anion [(PO_4_)W_12_O_36_]^3-^, three [C_6_H_12_N_5_O]^+^ cations and six water molecules of crystallisation. (Fig. 1). The anion in the title compound is of the well-known α-Keggin type consisting of four groups of W_3_O_10_ units. Each WO_6_ octahedron in such a unit shares edges with its neighbours. Four W_3_O_10_ units are linked together *via* corner-sharing WO_6_ octahedra to form a cage with a P atom located in the tetrahedrally surrounded centre. There are four kinds of oxygen atoms in the heteropolyanion, *viz*. four O atoms (O1c, O5c, O9c, O13c) that are bonded to the P atom and to three W atoms, twelve corner-sharing atoms Oc atom that bridge the different W_3_O_13_ units, twelve edge-sharing atoms Ob that bridge within the W_3_O_13_ units, and twelve terminal oxygen atoms Ot. In the organic cation, the 1*H*-tetrazole ring and the methylcarbon atom lie approximately in the same plane and the morpholine ring is in a chair configuration. In (I), all bond lengths and angles are normal and comparable with those observed in the pseudopolymorph with 5 crystal water molecules (Nikpour *et al.*, 2010)). The three organic cations in (I) show only minor differences with respect to bond lengths and angles.

The molecular entities are linked together *via* an extensive network of N—H···O, N—H···N, O—H···O and O—H···N hydrogen bonding interactions (Fig. 2). The charge-compensating cations [C_6_H_12_N_5_O]^+^ can be considered as the space-filling structural subunits and are connected to one side of the α-[(PO_4_)W_12_O_36_]^3-^ anion by the aforementioned multiple hydrogen-bonding interactions. Since [C_6_H_12_N_5_O]^+^ cations lie at one side of the anion, the inorganic anions are well-separated by [C_6_H_12_N_5_O]^+^ cations and by additional water molecules of crystallisation.

In recent years, there has been increasing interest in the experimental and theoretical study of water clusters (H_2_O)*_n_* because these water assemblies might provide an insight into some of the unexplained properties of bulk water, namely into the processes that occur at the ice-liquid, ice-air, and liquid-air interfaces, and into the nature of water-water and water-solute interactions (Aghabozorg *et al.* 2010). In the network of (I), six uncoordinated water molecules increase the number of O—H···O hydrogen bonds and thus lead to the formation of (H_2_O)_∞_ clusters. Indeed, these units were found to act as a 'supramolecular glue' in the aggregation of [C_6_H_12_N_5_O]_3_[W_12_(PO_4_)O_36_]^.^6H_2_O and hence support the consolidation of the three-dimensional network.

### Experimental

A solution of ((1*H*-tetrazole-5-yl)methyl)morpholine (0.14 g, 0.82 mmol) in 30 ml of distilled water was added with vigorous stirring to a solution of α-H_3_[(PO_4_)W_12_O_36_]^.^21H_2_O (0.50 g, 0.27 mmol) in 25 ml of distilled water. A colorless precipitate was formed after five hours. The solid was filtered off, washed with DMF and dried at room temperature. The precipitate was then re-dissolved in acetonitrile and the solution was cooled to ambient temperature; colorless block-shaped crystals were obtained, filtered off, washed several times with distilled water, and dried in air (yield 30% based on W) and characterized by spectroscopy and X-ray crystallography methods. ^1^H NMR in D_2_O:d 2.65 (t, 4H, (CH_2_)_2_N), 3.80 (t,4*H*, (CH_2_)_2_O), 4.15 (s, 2H, CH_2_-(N(CH_2_)_2_)).Anal. calcd. for C_18_H_45_N_15_O_49_PW_12_:*C*, 6.19; H, 1.30; N, 6.02; P, 0.90; W, 63.20. Found: C, 6.41; H, 1.41; N, 5.88; P, 0.85; W, 63.00.

### Refinement

Only heavy atoms (P and W) were refined anisotropically. Refinement in anisotropic approximation for all atoms was unstable due to the limited scattering powder of the crystal and absorption effects which could not be completely corrected. The highest peak and the deepest hole in the final Fourier map are 0.86 Å and 0.91 Å away from atoms W8 and W3, respectively.

Positions of hydrogen atoms were calculated. All hydrogen atoms were treated in the riding model approximation with the *U*_iso_(H) parameters equal to 1.2 *U*_eq_(Ci), where *U*_eq_(C) are the equivalent temperature factors of the atoms to which corresponding H atoms are bonded.

### Crystal data

**Table:**

(C_6_H_12_N_5_O)_3_[W_12_(PO_4_)O_36_]·6H_2_O	*F*(000) = 6224
*M**_r_* = 3495.88	*D*_x_ = 3.906 Mg m^−^^3^
Orthorhombic, *P*2_1_2_1_2_1_	Mo *K*α radiation, λ = 0.71073 Å
Hall symbol: P 2ac 2ab	Cell parameters from 846 reflections
*a* = 14.616 (3) Å	θ = 2.4–24.2°
*b* = 15.213 (3) Å	µ = 23.27 mm^−^^1^
*c* = 26.735 (6) Å	*T* = 100 K
*V* = 5944 (2) Å^3^	Plate, colourless
*Z* = 4	0.12 × 0.11 × 0.06 mm

### Data collection

**Table:**

Bruker APEXII CCD area-detector diffractometer	12321 independent reflections
Radiation source: fine-focus sealed tube	9617 reflections with *I* > 2σ(*I*)
graphite	*R*_int_ = 0.159
ω scans	θ_max_ = 26.5°, θ_min_ = 1.5°
Absorption correction: multi-scan (*SADABS*; Bruker, 2005)	*h* = −18→18
*T*_min_ = 0.078, *T*_max_ = 0.246	*k* = −19→19
59635 measured reflections	*l* = −33→33

### Refinement

**Table:**

Refinement on *F*^2^	Secondary atom site location: difference Fourier map
Least-squares matrix: full	Hydrogen site location: inferred from neighbouring sites
*R*[*F*^2^ > 2σ(*F*^2^)] = 0.052	H-atom parameters constrained
*wR*(*F*^2^) = 0.104	*w* = 1/[σ^2^(*F*_o_^2^) + (0.015*P*)^2^] where *P* = (*F*_o_^2^ + 2*F*_c_^2^)/3
*S* = 1.00	(Δ/σ)_max_ = 0.001
12321 reflections	Δρ_max_ = 2.80 e Å^−^^3^
446 parameters	Δρ_min_ = −2.72 e Å^−^^3^
6 restraints	Absolute structure: Flack (1983), 5544 Friedel pairs
Primary atom site location: structure-invariant direct methods	Flack parameter: −0.041 (19)

### Special details

**Table:**

Geometry. All e.s.d.'s (except the e.s.d. in the dihedral angle between two l.s. planes) are estimated using the full covariance matrix. The cell e.s.d.'s are taken into account individually in the estimation of e.s.d.'s in distances, angles and torsion angles; correlations between e.s.d.'s in cell parameters are only used when they are defined by crystal symmetry. An approximate (isotropic) treatment of cell e.s.d.'s is used for estimating e.s.d.'s involving l.s. planes.
Refinement. Refinement of *F*^2^ against ALL reflections. The weighted *R*-factor *wR* and goodness of fit *S* are based on *F*^2^, conventional *R*-factors *R* are based on *F*, with *F* set to zero for negative *F*^2^. The threshold expression of *F*^2^ > σ(*F*^2^) is used only for calculating *R*-factors(gt) *etc*. and is not relevant to the choice of reflections for refinement. *R*-factors based on *F*^2^ are statistically about twice as large as those based on *F*, and *R*- factors based on ALL data will be even larger.

### Fractional atomic coordinates and isotropic or equivalent isotropic displacement parameters (Å^2^)

**Table:**

	*x*	*y*	*z*	*U*_iso_*/*U*_eq_	
W1	0.13685 (6)	0.08083 (6)	0.78414 (3)	0.0071 (2)	
W2	0.12581 (6)	−0.11475 (6)	0.84527 (3)	0.00680 (19)	
W3	−0.01178 (6)	0.05564 (6)	0.88273 (3)	0.00663 (19)	
W4	0.23993 (6)	0.27465 (6)	0.84686 (3)	0.0063 (2)	
W5	0.32328 (6)	0.25584 (6)	0.96592 (3)	0.00742 (19)	
W6	0.09215 (6)	0.24836 (6)	0.94429 (3)	0.00671 (19)	
W7	0.38668 (6)	0.08881 (6)	0.80600 (3)	0.0069 (2)	
W8	0.46950 (6)	0.07104 (6)	0.92481 (3)	0.0077 (2)	
W9	0.37534 (7)	−0.10728 (6)	0.86647 (3)	0.0078 (2)	
W10	0.21725 (7)	−0.13462 (6)	0.97307 (3)	0.0076 (2)	
W11	0.08109 (6)	0.03551 (6)	1.01029 (3)	0.0071 (2)	
W12	0.31213 (6)	0.04266 (6)	1.03168 (3)	0.0075 (2)	
P1	0.2295 (4)	0.0669 (4)	0.9062 (2)	0.0048 (11)	
O1B	0.1641 (10)	0.1949 (9)	0.8099 (5)	0.004 (3)*	
O2B	0.2655 (11)	0.0633 (9)	0.7824 (5)	0.010 (3)*	
O3B	0.2554 (11)	−0.1191 (10)	0.8382 (6)	0.015 (4)*	
O4B	0.1447 (10)	−0.1393 (10)	0.9133 (5)	0.009 (3)*	
O5B	0.0152 (10)	0.0215 (9)	0.9489 (5)	0.006 (3)*	
O6B	0.0257 (11)	0.1702 (9)	0.9025 (5)	0.009 (3)*	
O7B	0.3371 (11)	0.1990 (10)	0.8259 (6)	0.011 (4)*	
O8B	0.4150 (11)	0.1809 (9)	0.9367 (5)	0.009 (3)*	
O9B	0.3045 (11)	0.1634 (9)	1.0121 (5)	0.007 (3)*	
O10B	0.0901 (11)	0.1564 (10)	0.9929 (6)	0.013 (4)*	
O11B	0.4062 (11)	0.0320 (10)	0.9827 (5)	0.012 (3)*	
O12B	0.3195 (10)	−0.1331 (9)	0.9280 (5)	0.008 (3)*	
O1C	0.1542 (10)	0.0355 (9)	0.8695 (5)	0.006 (3)*	
O2C	0.1237 (10)	−0.0454 (9)	0.7853 (5)	0.009 (3)*	
O3C	0.0181 (10)	0.0886 (9)	0.8151 (5)	0.006 (3)*	
O4C	0.0105 (10)	−0.0617 (9)	0.8616 (5)	0.009 (3)*	
O5C	0.2234 (11)	0.1677 (9)	0.9125 (5)	0.010 (4)*	
O6C	0.1364 (11)	0.3072 (9)	0.8851 (5)	0.008 (3)*	
O7C	0.3141 (10)	0.3122 (9)	0.9011 (5)	0.002 (3)*	
O8C	0.1998 (10)	0.2924 (9)	0.9769 (5)	0.004 (3)*	
O9C	0.3222 (11)	0.0400 (10)	0.8850 (5)	0.009 (3)*	
O10C	0.4081 (11)	−0.0347 (10)	0.8101 (5)	0.012 (3)*	
O11C	0.4790 (11)	0.1034 (10)	0.8545 (5)	0.012 (3)*	
O12C	0.4726 (12)	−0.0506 (11)	0.9018 (6)	0.017 (4)*	
O13C	0.2162 (10)	0.0225 (9)	0.9574 (5)	0.004 (3)*	
O14C	0.2926 (10)	−0.0796 (9)	1.0242 (5)	0.005 (3)*	
O15C	0.1137 (11)	−0.0849 (10)	1.0077 (5)	0.012 (3)*	
O16C	0.1849 (11)	0.0503 (10)	1.0520 (6)	0.014 (4)*	
O1T	0.1113 (11)	0.1009 (9)	0.7229 (5)	0.008 (3)*	
O2T	0.0886 (12)	−0.2120 (11)	0.8230 (6)	0.021 (4)*	
O3T	−0.1262 (11)	0.0627 (10)	0.8820 (5)	0.009 (3)*	
O4T	0.2503 (11)	0.3612 (9)	0.8069 (6)	0.011 (3)*	
O5T	0.3826 (11)	0.3329 (10)	0.9988 (6)	0.013 (4)*	
O6T	0.0161 (12)	0.3183 (10)	0.9630 (6)	0.015 (4)*	
O7T	0.4435 (10)	0.1134 (10)	0.7519 (5)	0.008 (3)*	
O8T	0.5799 (10)	0.0855 (10)	0.9435 (5)	0.009 (3)*	
O9T	0.4278 (10)	−0.2028 (9)	0.8515 (5)	0.008 (3)*	
O10T	0.2142 (10)	−0.2426 (9)	0.9929 (5)	0.007 (3)*	
O11T	−0.0015 (10)	0.0336 (9)	1.0544 (5)	0.007 (3)*	
O12T	0.3661 (11)	0.0419 (10)	1.0867 (5)	0.010 (3)*	
O1	−0.0329 (11)	0.6253 (10)	0.9033 (6)	0.014 (4)*	
O2	0.3910 (12)	0.1840 (10)	0.6455 (6)	0.014 (4)*	
O3	0.7992 (11)	0.3157 (10)	0.8498 (6)	0.013 (4)*	
N1A	0.2428 (14)	0.5669 (13)	1.0482 (7)	0.016 (5)*	
N2A	0.3121 (13)	0.6174 (12)	1.0671 (6)	0.010 (4)*	
N3A	0.3715 (13)	0.6348 (12)	1.0333 (7)	0.012 (4)*	
N4A	0.3446 (13)	0.5980 (12)	0.9905 (7)	0.014 (4)*	
H4A	0.3725	0.6005	0.9614	0.017*	
N5A	0.1426 (13)	0.5627 (13)	0.9376 (7)	0.014 (4)*	
H5A	0.1673	0.5989	0.9147	0.017*	
N1B	0.1622 (12)	0.4400 (11)	0.7087 (6)	0.006 (4)*	
N2B	0.1362 (15)	0.4894 (13)	0.7487 (7)	0.019 (5)*	
N3B	0.0718 (14)	0.4460 (13)	0.7736 (7)	0.017 (5)*	
N4B	0.0632 (13)	0.3681 (12)	0.7506 (6)	0.009 (4)*	
H4B	0.0263	0.3256	0.7601	0.010*	
N5B	0.2227 (13)	0.2763 (12)	0.6584 (7)	0.010 (4)*	
H5B	0.2237	0.3313	0.6460	0.012*	
N1C	0.4485 (14)	0.3858 (13)	0.7645 (7)	0.015 (5)*	
N2C	0.4037 (14)	0.4569 (13)	0.7477 (7)	0.015 (4)*	
N3C	0.3909 (14)	0.5159 (13)	0.7846 (7)	0.017 (5)*	
N4C	0.4281 (14)	0.4820 (13)	0.8246 (7)	0.017 (5)*	
H4C	0.4293	0.5058	0.8546	0.020*	
N5C	0.6017 (12)	0.3217 (11)	0.8337 (6)	0.005 (4)*	
H5C	0.6146	0.3135	0.8023	0.006*	
C1A	0.2647 (16)	0.5557 (14)	1.0014 (8)	0.013 (5)*	
C2A	0.2074 (16)	0.5054 (14)	0.9619 (8)	0.012 (5)*	
H2A	0.2489	0.4797	0.9365	0.014*	
H2B	0.1742	0.4566	0.9784	0.014*	
C3A	0.0767 (16)	0.6076 (15)	0.9709 (9)	0.014 (5)*	
H3A	0.1105	0.6373	0.9981	0.017*	
H3B	0.0358	0.5633	0.9863	0.017*	
C4A	0.0184 (18)	0.6757 (15)	0.9428 (8)	0.016 (5)*	
H4D	−0.0247	0.7050	0.9660	0.019*	
H4E	0.0579	0.7210	0.9273	0.019*	
C5A	0.0289 (14)	0.5823 (13)	0.8701 (7)	0.004 (4)*	
H5D	0.0670	0.6270	0.8531	0.005*	
H5E	−0.0066	0.5507	0.8442	0.005*	
C6A	0.0924 (15)	0.5159 (13)	0.8974 (7)	0.004 (4)*	
H6A	0.0556	0.4676	0.9119	0.004*	
H6B	0.1364	0.4901	0.8733	0.004*	
C1B	0.1192 (16)	0.3644 (14)	0.7108 (8)	0.011 (5)*	
C2B	0.1251 (15)	0.2898 (13)	0.6748 (7)	0.006 (4)*	
H2C	0.0864	0.3024	0.6452	0.007*	
H2D	0.1020	0.2355	0.6908	0.007*	
C3B	0.2338 (17)	0.2013 (14)	0.6242 (9)	0.013 (5)*	
H3C	0.2175	0.1464	0.6420	0.016*	
H3D	0.1910	0.2080	0.5957	0.016*	
C4B	0.3270 (17)	0.1938 (16)	0.6050 (9)	0.017 (6)*	
H4F	0.3313	0.1422	0.5825	0.021*	
H4G	0.3424	0.2469	0.5853	0.021*	
C5B	0.3838 (16)	0.2549 (14)	0.6768 (8)	0.011 (5)*	
H5F	0.3981	0.3090	0.6578	0.013*	
H5G	0.4299	0.2491	0.7038	0.013*	
C6B	0.2899 (14)	0.2648 (13)	0.7004 (7)	0.006 (5)*	
H6C	0.2746	0.2118	0.7202	0.007*	
H6D	0.2886	0.3165	0.7228	0.007*	
C1C	0.4646 (16)	0.4033 (15)	0.8114 (8)	0.013 (5)*	
C2C	0.5061 (16)	0.3423 (15)	0.8466 (8)	0.013 (5)*	
H2E	0.4701	0.2872	0.8472	0.016*	
H2F	0.5040	0.3682	0.8806	0.016*	
C3C	0.6655 (15)	0.3995 (14)	0.8253 (8)	0.007 (5)*	
H3E	0.6405	0.4372	0.7983	0.008*	
H3F	0.6691	0.4351	0.8562	0.008*	
C4C	0.7572 (18)	0.3700 (16)	0.8114 (9)	0.019 (6)*	
H4H	0.7536	0.3361	0.7799	0.022*	
H4I	0.7964	0.4220	0.8052	0.022*	
C5C	0.7445 (17)	0.2421 (15)	0.8584 (9)	0.015 (5)*	
H5H	0.7725	0.2068	0.8855	0.018*	
H5I	0.7439	0.2054	0.8278	0.018*	
C6C	0.6498 (17)	0.2625 (16)	0.8723 (8)	0.015 (5)*	
H6E	0.6495	0.2922	0.9053	0.019*	
H6F	0.6150	0.2069	0.8757	0.019*	
O1W	0.9409 (11)	0.2541 (10)	0.7904 (5)	0.013 (4)*	
H1W	0.9675	0.2096	0.8030	0.016*	
H2W	0.8972	0.2730	0.8085	0.016*	
O2W	0.4266 (11)	0.6202 (10)	0.8979 (5)	0.014 (4)*	
H35	0.4805	0.6109	0.9087	0.017*	
H36	0.4465	0.6690	0.8867	0.017*	
O3W	0.3234 (12)	0.4705 (11)	0.6534 (7)	0.027 (4)*	
H5W	0.3264	0.4896	0.6236	0.033*	
H6W	0.3570	0.4951	0.6752	0.033*	
O4W	0.6176 (14)	0.2121 (12)	0.7543 (6)	0.029 (5)*	
H7W	0.5676	0.1836	0.7536	0.035*	
H8W	0.6645	0.1847	0.7436	0.035*	
O5W	0.2453 (12)	0.6697 (10)	0.8761 (6)	0.018 (4)*	
H9W	0.2960	0.6448	0.8824	0.022*	
H10W	0.2317	0.6666	0.8452	0.022*	
O6W	0.2054 (12)	0.6585 (11)	0.7745 (6)	0.020 (4)*	
H71	0.1806	0.6207	0.7554	0.024*	
H72	0.1714	0.7033	0.7707	0.024*	

### Atomic displacement parameters (Å^2^)

**Table:**

	*U*^11^	*U*^22^	*U*^33^	*U*^12^	*U*^13^	*U*^23^
W1	0.0073 (5)	0.0071 (4)	0.0068 (4)	−0.0005 (4)	−0.0002 (4)	−0.0001 (4)
W2	0.0071 (5)	0.0054 (4)	0.0079 (4)	0.0006 (4)	−0.0003 (4)	−0.0009 (4)
W3	0.0049 (5)	0.0065 (4)	0.0085 (4)	0.0006 (4)	0.0000 (4)	−0.0006 (4)
W4	0.0061 (5)	0.0051 (4)	0.0077 (5)	−0.0002 (4)	0.0001 (4)	0.0006 (4)
W5	0.0080 (5)	0.0055 (4)	0.0088 (5)	−0.0005 (4)	−0.0015 (4)	0.0001 (4)
W6	0.0060 (5)	0.0051 (4)	0.0091 (4)	0.0001 (4)	0.0014 (4)	−0.0003 (4)
W7	0.0050 (5)	0.0068 (4)	0.0090 (4)	0.0005 (4)	0.0007 (4)	0.0007 (4)
W8	0.0061 (5)	0.0076 (4)	0.0094 (4)	−0.0004 (4)	−0.0014 (4)	0.0007 (4)
W9	0.0058 (5)	0.0061 (4)	0.0114 (5)	0.0012 (4)	0.0015 (4)	−0.0002 (4)
W10	0.0088 (5)	0.0052 (4)	0.0088 (5)	−0.0003 (4)	−0.0004 (4)	0.0008 (4)
W11	0.0070 (5)	0.0068 (4)	0.0076 (5)	0.0000 (4)	0.0010 (4)	0.0009 (4)
W12	0.0089 (5)	0.0073 (4)	0.0064 (5)	0.0002 (4)	−0.0008 (4)	0.0008 (4)
P1	0.0043 (18)	0.0060 (17)	0.0042 (17)	−0.0007 (15)	−0.0014 (15)	0.0003 (15)

### Geometric parameters (Å, °)

**Table:**

W1—O1T	1.706 (14)	N1A—C1A	1.30 (3)
W1—O2B	1.900 (16)	N1A—N2A	1.37 (3)
W1—O1B	1.909 (14)	N2A—N3A	1.28 (2)
W1—O3C	1.926 (15)	N3A—N4A	1.33 (2)
W1—O2C	1.930 (14)	N4A—C1A	1.37 (3)
W1—O1C	2.396 (14)	N4A—H4A	0.8800
W2—O2T	1.685 (17)	N5A—C2A	1.44 (3)
W2—O4B	1.878 (14)	N5A—C6A	1.48 (3)
W2—O3B	1.905 (17)	N5A—C3A	1.48 (3)
W2—O2C	1.920 (14)	N5A—H5A	0.8997
W2—O4C	1.919 (15)	N1B—C1B	1.31 (3)
W2—O1C	2.412 (14)	N1B—N2B	1.36 (3)
W3—O3T	1.676 (15)	N2B—N3B	1.33 (3)
W3—O5B	1.886 (14)	N3B—N4B	1.34 (3)
W3—O4C	1.900 (14)	N4B—C1B	1.34 (3)
W3—O6B	1.902 (15)	N4B—H4B	0.8800
W3—O3C	1.925 (13)	N5B—C3B	1.47 (3)
W3—O1C	2.471 (15)	N5B—C6B	1.50 (3)
W4—O4T	1.703 (15)	N5B—C2B	1.51 (3)
W4—O6C	1.893 (15)	N5B—H5B	0.9000
W4—O7C	1.899 (14)	N1C—C1C	1.30 (3)
W4—O7B	1.912 (16)	N1C—N2C	1.34 (3)
W4—O1B	1.917 (14)	N2C—N3C	1.35 (3)
W4—O5C	2.405 (14)	N3C—N4C	1.31 (3)
W5—O5T	1.702 (16)	N4C—C1C	1.36 (3)
W5—O9B	1.892 (14)	N4C—H4C	0.8800
W5—O8C	1.911 (15)	N5C—C2C	1.47 (3)
W5—O8B	1.925 (15)	N5C—C3C	1.52 (3)
W5—O7C	1.938 (13)	N5C—C6C	1.54 (3)
W5—O5C	2.444 (15)	N5C—H5C	0.8698
W6—O6T	1.619 (16)	C1A—C2A	1.55 (3)
W6—O6B	1.898 (15)	C2A—H2A	0.9900
W6—O10B	1.910 (15)	C2A—H2B	0.9900
W6—O8C	1.919 (14)	C3A—C4A	1.54 (3)
W6—O6C	1.928 (14)	C3A—H3A	0.9900
W6—O5C	2.430 (15)	C3A—H3B	0.9900
W7—O7T	1.710 (14)	C4A—H4D	0.9900
W7—O11C	1.884 (15)	C4A—H4E	0.9900
W7—O7B	1.902 (15)	C5A—C6A	1.55 (3)
W7—O10C	1.908 (15)	C5A—H5D	0.9900
W7—O2B	1.920 (15)	C5A—H5E	0.9900
W7—O9C	2.428 (14)	C6A—H6A	0.9900
W8—O8T	1.704 (15)	C6A—H6B	0.9900
W8—O8B	1.879 (15)	C1B—C2B	1.49 (3)
W8—O11B	1.900 (15)	C2B—H2C	0.9900
W8—O12C	1.950 (16)	C2B—H2D	0.9900
W8—O11C	1.949 (14)	C3B—C4B	1.46 (3)
W8—O9C	2.448 (15)	C3B—H3C	0.9900
W9—O9T	1.691 (14)	C3B—H3D	0.9900
W9—O12B	1.878 (15)	C4B—H4F	0.9900
W9—O12C	1.913 (17)	C4B—H4G	0.9900
W9—O3B	1.918 (17)	C5B—C6B	1.52 (3)
W9—O10C	1.928 (15)	C5B—H5F	0.9900
W9—O9C	2.423 (15)	C5B—H5G	0.9900
W10—O10T	1.727 (14)	C6B—H6C	0.9900
W10—O12B	1.920 (15)	C6B—H6D	0.9900
W10—O4B	1.918 (14)	C1C—C2C	1.46 (3)
W10—O15C	1.929 (15)	C2C—H2E	0.9900
W10—O14C	1.944 (14)	C2C—H2F	0.9900
W10—O13C	2.427 (13)	C3C—C4C	1.46 (3)
W11—O11T	1.687 (14)	C3C—H3E	0.9900
W11—O15C	1.895 (15)	C3C—H3F	0.9900
W11—O16C	1.897 (16)	C4C—H4H	0.9900
W11—O10B	1.901 (15)	C4C—H4I	0.9900
W11—O5B	1.914 (14)	C5C—C6C	1.47 (3)
W11—O13C	2.437 (14)	C5C—H5H	0.9900
W12—O12T	1.668 (14)	C5C—H5I	0.9900
W12—O14C	1.892 (13)	C6C—H6E	0.9900
W12—O11B	1.904 (15)	C6C—H6F	0.9900
W12—O9B	1.913 (14)	O1W—H1W	0.8498
W12—O16C	1.941 (16)	O1W—H2W	0.8501
W12—O13C	2.451 (14)	O2W—H35	0.8500
P1—O9C	1.525 (16)	O2W—H36	0.8500
P1—O13C	1.538 (14)	O3W—H5W	0.8498
P1—O5C	1.545 (15)	O3W—H6W	0.8501
P1—O1C	1.550 (15)	O4W—H7W	0.8500
O1—C5A	1.43 (2)	O4W—H8W	0.8500
O1—C4A	1.51 (3)	O5W—H9W	0.8499
O2—C5B	1.37 (2)	O5W—H10W	0.8500
O2—C4B	1.44 (3)	O6W—H71	0.8500
O3—C5C	1.40 (3)	O6W—H72	0.8499
O3—C4C	1.45 (3)		
			
O1T—W1—O2B	102.6 (7)	W2—O4B—W10	151.7 (9)
O1T—W1—O1B	103.2 (6)	W3—O5B—W11	151.5 (8)
O2B—W1—O1B	86.0 (6)	W6—O6B—W3	152.2 (9)
O1T—W1—O3C	101.8 (7)	W7—O7B—W4	153.5 (9)
O2B—W1—O3C	155.6 (6)	W8—O8B—W5	153.0 (9)
O1B—W1—O3C	88.7 (6)	W5—O9B—W12	152.2 (8)
O1T—W1—O2C	99.9 (6)	W11—O10B—W6	151.1 (9)
O2B—W1—O2C	87.7 (6)	W8—O11B—W12	152.3 (9)
O1B—W1—O2C	156.8 (6)	W9—O12B—W10	152.9 (9)
O3C—W1—O2C	87.9 (6)	P1—O1C—W1	126.1 (8)
O1T—W1—O1C	171.0 (6)	P1—O1C—W2	125.7 (8)
O2B—W1—O1C	83.0 (6)	W1—O1C—W2	90.0 (5)
O1B—W1—O1C	84.0 (5)	P1—O1C—W3	124.6 (8)
O3C—W1—O1C	72.7 (5)	W1—O1C—W3	89.8 (5)
O2C—W1—O1C	73.1 (5)	W2—O1C—W3	89.3 (5)
O2T—W2—O4B	102.4 (7)	W2—O2C—W1	124.0 (7)
O2T—W2—O3B	104.8 (8)	W1—O3C—W3	126.3 (7)
O4B—W2—O3B	86.7 (7)	W3—O4C—W2	127.8 (8)
O2T—W2—O2C	100.5 (7)	P1—O5C—W4	125.9 (8)
O4B—W2—O2C	157.1 (6)	P1—O5C—W6	125.8 (9)
O3B—W2—O2C	87.3 (7)	W4—O5C—W6	89.6 (5)
O2T—W2—O4C	99.5 (8)	P1—O5C—W5	125.0 (9)
O4B—W2—O4C	89.6 (6)	W4—O5C—W5	89.7 (5)
O3B—W2—O4C	155.6 (6)	W6—O5C—W5	89.4 (5)
O2C—W2—O4C	86.8 (6)	W4—O6C—W6	126.2 (8)
O2T—W2—O1C	169.3 (7)	W4—O7C—W5	126.1 (7)
O4B—W2—O1C	84.5 (6)	W5—O8C—W6	127.1 (7)
O3B—W2—O1C	83.6 (6)	P1—O9C—W9	127.5 (9)
O2C—W2—O1C	72.9 (5)	P1—O9C—W7	125.9 (8)
O4C—W2—O1C	72.0 (6)	W9—O9C—W7	88.9 (5)
O3T—W3—O5B	103.7 (7)	P1—O9C—W8	124.6 (8)
O3T—W3—O4C	103.1 (7)	W9—O9C—W8	89.1 (5)
O5B—W3—O4C	89.1 (6)	W7—O9C—W8	88.7 (5)
O3T—W3—O6B	103.4 (7)	W7—O10C—W9	124.6 (8)
O5B—W3—O6B	86.1 (6)	W7—O11C—W8	125.6 (8)
O4C—W3—O6B	153.4 (7)	W9—O12C—W8	124.5 (9)
O3T—W3—O3C	101.5 (7)	P1—O13C—W10	125.8 (8)
O5B—W3—O3C	154.8 (6)	P1—O13C—W11	125.7 (8)
O4C—W3—O3C	85.8 (6)	W10—O13C—W11	89.1 (5)
O6B—W3—O3C	87.5 (6)	P1—O13C—W12	126.4 (8)
O3T—W3—O1C	170.4 (6)	W10—O13C—W12	88.8 (4)
O5B—W3—O1C	84.0 (6)	W11—O13C—W12	89.0 (4)
O4C—W3—O1C	70.9 (6)	W12—O14C—W10	125.7 (7)
O6B—W3—O1C	82.6 (6)	W11—O15C—W10	126.4 (8)
O3C—W3—O1C	71.0 (5)	W11—O16C—W12	126.5 (8)
O4T—W4—O6C	102.0 (7)	C5A—O1—C4A	110.7 (17)
O4T—W4—O7C	101.3 (7)	C5B—O2—C4B	109.3 (17)
O6C—W4—O7C	88.0 (6)	C5C—O3—C4C	109.4 (18)
O4T—W4—O7B	102.4 (7)	C1A—N1A—N2A	104.3 (19)
O6C—W4—O7B	155.5 (6)	N3A—N2A—N1A	110.9 (17)
O7C—W4—O7B	88.9 (6)	N2A—N3A—N4A	108.6 (18)
O4T—W4—O1B	102.5 (7)	N3A—N4A—C1A	105.5 (18)
O6C—W4—O1B	89.0 (6)	N3A—N4A—H4A	127.3
O7C—W4—O1B	156.1 (6)	C1A—N4A—H4A	127.3
O7B—W4—O1B	84.1 (6)	C2A—N5A—C6A	111.2 (17)
O4T—W4—O5C	171.9 (6)	C2A—N5A—C3A	115.9 (18)
O6C—W4—O5C	72.7 (6)	C6A—N5A—C3A	109.6 (18)
O7C—W4—O5C	72.8 (5)	C2A—N5A—H5A	114.4
O7B—W4—O5C	83.2 (6)	C6A—N5A—H5A	89.9
O1B—W4—O5C	83.7 (5)	C3A—N5A—H5A	112.8
O5T—W5—O9B	104.5 (7)	C1B—N1B—N2B	108.5 (18)
O5T—W5—O8C	101.7 (7)	N3B—N2B—N1B	108.5 (18)
O9B—W5—O8C	88.8 (6)	N2B—N3B—N4B	106.0 (18)
O5T—W5—O8B	105.2 (7)	N3B—N4B—C1B	110.1 (19)
O9B—W5—O8B	85.7 (6)	N3B—N4B—H4B	124.9
O8C—W5—O8B	153.1 (6)	C1B—N4B—H4B	124.9
O5T—W5—O7C	101.1 (6)	C3B—N5B—C6B	107.6 (17)
O9B—W5—O7C	154.5 (6)	C3B—N5B—C2B	113.0 (17)
O8C—W5—O7C	86.7 (6)	C6B—N5B—C2B	114.7 (16)
O8B—W5—O7C	87.0 (6)	C3B—N5B—H5B	119.4
O5T—W5—O5C	169.7 (6)	C6B—N5B—H5B	111.8
O9B—W5—O5C	83.5 (6)	C2B—N5B—H5B	89.8
O8C—W5—O5C	71.6 (6)	C1C—N1C—N2C	104.1 (19)
O8B—W5—O5C	81.6 (6)	N1C—N2C—N3C	111.2 (18)
O7C—W5—O5C	71.3 (5)	N4C—N3C—N2C	106.2 (19)
O6T—W6—O6B	104.1 (8)	N3C—N4C—C1C	107.4 (19)
O6T—W6—O10B	105.1 (7)	N3C—N4C—H4C	126.3
O6B—W6—O10B	86.2 (6)	C1C—N4C—H4C	126.3
O6T—W6—O8C	101.1 (7)	C2C—N5C—C3C	116.8 (16)
O6B—W6—O8C	154.8 (6)	C2C—N5C—C6C	113.6 (17)
O10B—W6—O8C	87.7 (6)	C3C—N5C—C6C	105.9 (16)
O6T—W6—O6C	100.3 (7)	C2C—N5C—H5C	117.6
O6B—W6—O6C	88.8 (6)	C3C—N5C—H5C	80.4
O10B—W6—O6C	154.6 (7)	C6C—N5C—H5C	117.6
O8C—W6—O6C	86.3 (6)	N1A—C1A—N4A	111 (2)
O6T—W6—O5C	169.2 (7)	N1A—C1A—C2A	126 (2)
O6B—W6—O5C	83.2 (6)	N4A—C1A—C2A	123 (2)
O10B—W6—O5C	83.1 (6)	N5A—C2A—C1A	111.3 (18)
O8C—W6—O5C	71.8 (5)	N5A—C2A—H2A	109.4
O6C—W6—O5C	71.5 (6)	C1A—C2A—H2A	109.4
O7T—W7—O11C	102.0 (7)	N5A—C2A—H2B	109.4
O7T—W7—O7B	103.2 (7)	C1A—C2A—H2B	109.4
O11C—W7—O7B	88.6 (7)	H2A—C2A—H2B	108.0
O7T—W7—O10C	100.6 (7)	N5A—C3A—C4A	112.2 (18)
O11C—W7—O10C	87.6 (6)	N5A—C3A—H3A	109.2
O7B—W7—O10C	156.1 (7)	C4A—C3A—H3A	109.2
O7T—W7—O2B	102.4 (7)	N5A—C3A—H3B	109.2
O11C—W7—O2B	155.6 (6)	C4A—C3A—H3B	109.2
O7B—W7—O2B	85.4 (6)	H3A—C3A—H3B	107.9
O10C—W7—O2B	88.4 (6)	O1—C4A—C3A	106.0 (18)
O7T—W7—O9C	172.4 (6)	O1—C4A—H4D	110.5
O11C—W7—O9C	73.5 (6)	C3A—C4A—H4D	110.5
O7B—W7—O9C	83.0 (6)	O1—C4A—H4E	110.5
O10C—W7—O9C	73.3 (6)	C3A—C4A—H4E	110.5
O2B—W7—O9C	82.3 (6)	H4D—C4A—H4E	108.7
O8T—W8—O8B	103.7 (7)	O1—C5A—C6A	112.7 (16)
O8T—W8—O11B	105.2 (7)	O1—C5A—H5D	109.1
O8B—W8—O11B	86.2 (6)	C6A—C5A—H5D	109.1
O8T—W8—O12C	101.1 (7)	O1—C5A—H5E	109.1
O8B—W8—O12C	155.2 (7)	C6A—C5A—H5E	109.1
O11B—W8—O12C	88.4 (6)	H5D—C5A—H5E	107.8
O8T—W8—O11C	100.6 (7)	N5A—C6A—C5A	108.9 (16)
O8B—W8—O11C	88.2 (6)	N5A—C6A—H6A	109.9
O11B—W8—O11C	154.2 (7)	C5A—C6A—H6A	109.9
O12C—W8—O11C	86.2 (6)	N5A—C6A—H6B	109.9
O8T—W8—O9C	170.3 (6)	C5A—C6A—H6B	109.9
O8B—W8—O9C	82.7 (6)	H6A—C6A—H6B	108.3
O11B—W8—O9C	82.3 (6)	N1B—C1B—N4B	106.8 (19)
O12C—W8—O9C	72.6 (6)	N1B—C1B—C2B	128 (2)
O11C—W8—O9C	72.0 (6)	N4B—C1B—C2B	125 (2)
O9T—W9—O12B	102.9 (7)	C1B—C2B—N5B	110.3 (18)
O9T—W9—O12C	99.6 (7)	C1B—C2B—H2C	109.6
O12B—W9—O12C	89.1 (6)	N5B—C2B—H2C	109.6
O9T—W9—O3B	103.9 (7)	C1B—C2B—H2D	109.6
O12B—W9—O3B	85.9 (7)	N5B—C2B—H2D	109.6
O12C—W9—O3B	156.4 (7)	H2C—C2B—H2D	108.1
O9T—W9—O10C	101.2 (7)	C4B—C3B—N5B	112 (2)
O12B—W9—O10C	155.8 (6)	C4B—C3B—H3C	109.1
O12C—W9—O10C	86.7 (7)	N5B—C3B—H3C	109.1
O3B—W9—O10C	88.5 (7)	C4B—C3B—H3D	109.1
O9T—W9—O9C	171.3 (6)	N5B—C3B—H3D	109.1
O12B—W9—O9C	82.9 (6)	H3C—C3B—H3D	107.8
O12C—W9—O9C	73.8 (6)	O2—C4B—C3B	110.5 (19)
O3B—W9—O9C	82.8 (6)	O2—C4B—H4F	109.5
O10C—W9—O9C	73.1 (6)	C3B—C4B—H4F	109.5
O10T—W10—O12B	102.9 (6)	O2—C4B—H4G	109.5
O10T—W10—O4B	101.9 (6)	C3B—C4B—H4G	109.5
O12B—W10—O4B	84.7 (6)	H4F—C4B—H4G	108.1
O10T—W10—O15C	101.9 (7)	O2—C5B—C6B	113.7 (19)
O12B—W10—O15C	155.2 (6)	O2—C5B—H5F	108.8
O4B—W10—O15C	88.9 (6)	C6B—C5B—H5F	108.8
O10T—W10—O14C	102.1 (6)	O2—C5B—H5G	108.8
O12B—W10—O14C	89.7 (6)	C6B—C5B—H5G	108.8
O4B—W10—O14C	156.1 (6)	H5F—C5B—H5G	107.7
O15C—W10—O14C	86.5 (6)	N5B—C6B—C5B	107.0 (16)
O10T—W10—O13C	171.9 (6)	N5B—C6B—H6C	110.3
O12B—W10—O13C	83.4 (6)	C5B—C6B—H6C	110.3
O4B—W10—O13C	83.6 (6)	N5B—C6B—H6D	110.3
O15C—W10—O13C	72.1 (6)	C5B—C6B—H6D	110.3
O14C—W10—O13C	72.6 (5)	H6C—C6B—H6D	108.6
O11T—W11—O15C	100.9 (7)	N1C—C1C—N4C	111 (2)
O11T—W11—O16C	99.4 (7)	N1C—C1C—C2C	125 (2)
O15C—W11—O16C	86.3 (7)	N4C—C1C—C2C	124 (2)
O11T—W11—O10B	103.7 (7)	C1C—C2C—N5C	112.3 (19)
O15C—W11—O10B	155.4 (7)	C1C—C2C—H2E	109.1
O16C—W11—O10B	88.5 (7)	N5C—C2C—H2E	109.1
O11T—W11—O5B	103.7 (7)	C1C—C2C—H2F	109.1
O15C—W11—O5B	89.3 (6)	N5C—C2C—H2F	109.1
O16C—W11—O5B	156.9 (6)	H2E—C2C—H2F	107.9
O10B—W11—O5B	86.2 (6)	C4C—C3C—N5C	111.2 (18)
O11T—W11—O13C	169.7 (6)	C4C—C3C—H3E	109.4
O15C—W11—O13C	72.4 (6)	N5C—C3C—H3E	109.4
O16C—W11—O13C	72.7 (6)	C4C—C3C—H3F	109.4
O10B—W11—O13C	83.1 (6)	N5C—C3C—H3F	109.4
O5B—W11—O13C	84.3 (5)	H3E—C3C—H3F	108.0
O12T—W12—O14C	99.1 (7)	O3—C4C—C3C	112.5 (19)
O12T—W12—O11B	105.3 (7)	O3—C4C—H4H	109.1
O14C—W12—O11B	87.2 (6)	C3C—C4C—H4H	109.1
O12T—W12—O9B	106.0 (7)	O3—C4C—H4I	109.1
O14C—W12—O9B	154.9 (6)	C3C—C4C—H4I	109.1
O11B—W12—O9B	86.3 (6)	H4H—C4C—H4I	107.8
O12T—W12—O16C	101.9 (7)	O3—C5C—C6C	114 (2)
O14C—W12—O16C	86.8 (6)	O3—C5C—H5H	108.7
O11B—W12—O16C	152.8 (6)	C6C—C5C—H5H	108.7
O9B—W12—O16C	87.9 (7)	O3—C5C—H5I	108.7
O12T—W12—O13C	169.7 (6)	C6C—C5C—H5I	108.7
O14C—W12—O13C	72.8 (5)	H5H—C5C—H5I	107.6
O11B—W12—O13C	81.1 (6)	C5C—C6C—N5C	112.7 (19)
O9B—W12—O13C	82.2 (5)	C5C—C6C—H6E	109.1
O16C—W12—O13C	71.7 (6)	N5C—C6C—H6E	109.1
O9C—P1—O13C	109.0 (9)	C5C—C6C—H6F	109.1
O9C—P1—O5C	111.0 (9)	N5C—C6C—H6F	109.1
O13C—P1—O5C	109.3 (8)	H6E—C6C—H6F	107.8
O9C—P1—O1C	108.2 (8)	H1W—O1W—H2W	112.9
O13C—P1—O1C	109.8 (8)	H35—O2W—H36	87.1
O5C—P1—O1C	109.5 (9)	H5W—O3W—H6W	117.6
W1—O1B—W4	151.8 (8)	H7W—O4W—H8W	115.8
W1—O2B—W7	151.2 (8)	H9W—O5W—H10W	111.9
W2—O3B—W9	150.1 (9)	H71—O6W—H72	102.9
			
C1A—N1A—N2A—N3A	0(2)	N1B—C1B—C2B—N5B	41 (3)
N1A—N2A—N3A—N4A	1(2)	N4B—C1B—C2B—N5B	−143 (2)
N2A—N3A—N4A—C1A	−1(2)	C3B—N5B—C2B—C1B	177.8 (18)
C1B—N1B—N2B—N3B	−4(3)	C6B—N5B—C2B—C1B	54 (2)
N1B—N2B—N3B—N4B	4(2)	C6B—N5B—C3B—C4B	−58 (2)
N2B—N3B—N4B—C1B	−2(2)	C2B—N5B—C3B—C4B	174.7 (18)
C1C—N1C—N2C—N3C	−2(3)	C5B—O2—C4B—C3B	−58 (2)
N1C—N2C—N3C—N4C	0(3)	N5B—C3B—C4B—O2	59 (3)
N2C—N3C—N4C—C1C	1(3)	C4B—O2—C5B—C6B	61 (2)
N2A—N1A—C1A—N4A	−1(2)	C3B—N5B—C6B—C5B	55 (2)
N2A—N1A—C1A—C2A	−178 (2)	C2B—N5B—C6B—C5B	−178.3 (17)
N3A—N4A—C1A—N1A	1(3)	O2—C5B—C6B—N5B	−60 (2)
N3A—N4A—C1A—C2A	178.3 (19)	N2C—N1C—C1C—N4C	2(3)
C6A—N5A—C2A—C1A	176.0 (18)	N2C—N1C—C1C—C2C	175 (2)
C3A—N5A—C2A—C1A	−58 (3)	N3C—N4C—C1C—N1C	−2(3)
N1A—C1A—C2A—N5A	88 (3)	N3C—N4C—C1C—C2C	−175 (2)
N4A—C1A—C2A—N5A	−89 (3)	N1C—C1C—C2C—N5C	66 (3)
C2A—N5A—C3A—C4A	172.9 (19)	N4C—C1C—C2C—N5C	−122 (2)
C6A—N5A—C3A—C4A	−60 (2)	C3C—N5C—C2C—C1C	52 (2)
C5A—O1—C4A—C3A	−60 (2)	C6C—N5C—C2C—C1C	176.0 (18)
N5A—C3A—C4A—O1	61 (2)	C2C—N5C—C3C—C4C	−178.4 (19)
C4A—O1—C5A—C6A	60 (2)	C6C—N5C—C3C—C4C	54 (2)
C2A—N5A—C6A—C5A	−176.0 (17)	C5C—O3—C4C—C3C	59 (2)
C3A—N5A—C6A—C5A	55 (2)	N5C—C3C—C4C—O3	−60 (2)
O1—C5A—C6A—N5A	−56 (2)	C4C—O3—C5C—C6C	−56 (3)
N2B—N1B—C1B—N4B	3(2)	O3—C5C—C6C—N5C	55 (3)
N2B—N1B—C1B—C2B	179 (2)	C2C—N5C—C6C—C5C	179.4 (19)
N3B—N4B—C1B—N1B	0(3)	C3C—N5C—C6C—C5C	−51 (2)
N3B—N4B—C1B—C2B	−177 (2)		

### Hydrogen-bond geometry (Å, °)

**Table:**

*D*—H···*A*	*D*—H	H···*A*	*D*···*A*	*D*—H···*A*
N4A—H4A···O2W	0.88	1.90	2.77 (2)	173
N5A—H5A···O5W	0.90	1.88	2.76 (3)	166
N4B—H4B···O1W^i^	0.88	1.84	2.71 (2)	168
N5B—H5B···N2A^ii^	0.90	2.31	2.97 (2)	130
N4C—H4C···O2W	0.88	2.09	2.87 (3)	149
N5C—H5C···O4W	0.87	2.01	2.71 (2)	137
O1W—H1W···O3C^iii^	0.85	2.01	2.84 (2)	164
O1W—H2W···O3	0.85	1.92	2.77 (2)	180
O2W—H3W···O11T^iv^	0.85	2.43	2.87 (2)	113
O2W—H4W···O9T^v^	0.85	2.18	2.96 (2)	153
O2W—H4W···O2^vi^	0.85	2.54	3.06 (2)	121
O3W—H5W···N1A^ii^	0.85	2.41	3.03 (3)	130
O3W—H6W···N2C	0.85	2.14	2.79 (3)	134
O4W—H7W···O7T	0.85	2.11	2.96 (2)	180
O4W—H8W···O6W^vii^	0.85	2.00	2.82 (2)	161
O5W—H9W···O2W	0.85	1.99	2.82 (2)	164
O5W—H10W···O6W	0.85	1.93	2.78 (2)	178
O6W—H11W···N2B	0.85	2.11	2.85 (2)	146
O6W—H12W···O2T^v^	0.85	2.25	2.91 (2)	134
O6W—H12W···O1W^vi^	0.85	2.44	3.11 (2)	137

Symmetry codes:  (i) *x*−1, *y*, *z*; (ii) −*x*+1/2, −*y*+1, *z*−1/2; (iii) *x*+1, *y*, *z*; (iv) *x*+1/2, −*y*+1/2, −*z*+2; (v) *x*, *y*+1, *z*; (vi) −*x*+1, *y*+1/2, −*z*+3/2; (vii) −*x*+1, *y*−1/2, −*z*+3/2.
